# Psychological Correlates of Tinnitus Handicap: Associations with Personality Traits and Emotional Distress

**DOI:** 10.3390/jcm15155796

**Published:** 2026-07-24

**Authors:** Tomislav Sušac, Ivona Čarapina Zovko, Josip Lesko, Goran Šimić, Anamarija Mikulić, Boris Jelavić

**Affiliations:** 1 Department of Otorhinolaryngology, University Clinical Hospital Mostar, 88 000 Mostar, Bosnia and Herzegovina; tomislavsusac@gmail.com (T.S.); lesko.josip@gmail.com (J.L.); goransimic129@gmail.com (G.Š.); aleventic1@gmail.com (A.M.); slav.boris@tel.net.ba (B.J.); 2 Department of Psychology, Faculty of Humanities and Social Sciences, University of Mostar, 88 000 Mostar, Bosnia and Herzegovina; 3 School of Medicine, University of Mostar, 88 000 Mostar, Bosnia and Herzegovina

**Keywords:** tinnitus, handicap, depression, anxiety, stress, personality traits

## Abstract

**Background/Objectives**: Tinnitus is often a distressing and disabling condition that negatively affects individuals’ emotional well-being and quality of life. The aim of this study was to evaluate the relationship between perceived tinnitus handicap and sociodemographic and clinical characteristics, personality traits, and symptoms of depression, anxiety, and stress. **Methods:** Tinnitus patients with hearing loss (*N* = 127) underwent audiologic testing and completed the Tinnitus Handicap Inventory (THI), Depression Anxiety Stress Scales (DASS), and Eysenck Personality Questionnaire—Revised Short Form (EPQ-RS). Patient sociodemographic data (age, sex, and education level) and descriptive tinnitus characteristics were collected. The control group (*N* = 119) comprised tinnitus-free patients with hearing loss. **Results:** Patients experiencing catastrophic tinnitus reported significantly higher depression, anxiety, and stress scores compared to those with slight, mild, or moderate tinnitus grades. Education level was weakly negatively associated with THI scores. Conversely, weak-to-moderate positive relationships were observed for tinnitus loudness, pure tone average, the three DASS subscales, and the neuroticism subscale. Hierarchical multiple regression identified subjective tinnitus loudness, neuroticism, and total emotional distress (the total DASS score) as statistically significant predictors of the total THI score. Patients with tinnitus showed statistically significantly higher depression, anxiety, and stress subscale scores compared to tinnitus-free controls. **Conclusions:** The severity of tinnitus handicap is strongly correlated with subjective loudness, underlying neuroticism, and emotional distress. Patients with catastrophic tinnitus exhibit markedly higher levels of depression, anxiety, and stress. Consequently, clinical management of tinnitus must move beyond standard audiologic testing to include comprehensive psychological screening and targeted distress-reduction therapies.

## 1. Introduction

Tinnitus is a phantom perception of sound in the absence of an external acoustic stimulus. Tinnitus frequently co-occurs with sensorineural hearing loss and may also interfere with speech recognition in ways that are not fully captured by pure-tone thresholds alone; recent evidence has demonstrated associations between tinnitus intensity, speech-audiometry performance, and perceived tinnitus handicap [1]. Although tinnitus is primarily evaluated within an audiological and otological framework, systemic and metabolic factors may also contribute to its occurrence and severity, supporting a broader clinical assessment of affected patients [2].

Although tinnitus is often considered a benign auditory phenomenon, a substantial proportion of individuals experience it as a distressing and disabling condition that negatively affects emotional well-being and quality of life [3]. According to the neurophysiological model proposed by Jastreboff and Hazell, tinnitus-related distress develops through interactions between the auditory system and emotional processing, resulting in a self-perpetuating cycle in which negative emotional reactions reinforce the perception of tinnitus [3]. Accumulating evidence suggests that tinnitus is closely associated with psychological distress. A systematic review by Meijers et al. [4] found significant associations between tinnitus distress and depressive symptoms in 31 of 33 studies, with the prevalence of clinically significant depression ranging from 4.6% to 41.7%. Similarly, a large population-based cohort study demonstrated that individuals with tinnitus were significantly more likely to experience depression, anxiety, and somatization than those without tinnitus [5]. More recently, a meta-analysis by Jiang et al. [6] confirmed robust associations between tinnitus and depression, anxiety, stress, insomnia, and suicidal ideation, highlighting the importance of considering psychological factors in the assessment and management of tinnitus.

Beyond emotional symptoms, increasing attention has been directed toward personality traits as potential determinants of tinnitus-related distress. Among the major personality dimensions, neuroticism has consistently emerged as the strongest correlate of tinnitus handicap [7,8]. Previous findings suggest that relatively stable personality characteristics may influence the way individuals perceive, interpret, and cope with chronic tinnitus.

Although previous studies have established associations between tinnitus distress, emotional symptoms, and personality traits, these factors have often been investigated separately. Guided by a biopsychosocial understanding of tinnitus, we considered a simultaneous approach necessary because tinnitus handicap is likely to reflect the combined and partly overlapping influence of audiological characteristics, personality-related vulnerability, and emotional distress rather than the isolated effect of any single domain. Examining these factors within the same analytical framework allows their relative and independent contributions to tinnitus handicap to be assessed and helps determine whether psychological factors remain associated with tinnitus handicap after audiological characteristics are taken into account. In addition, geographical differences in tinnitus prevalence and severity have been reported across Europe, probably reflecting socioeconomic, environmental, occupational, and healthcare-related factors [9]. Consequently, evidence from Southeast European populations remains limited. To the best of our knowledge, no previous study conducted in Bosnia and Herzegovina has simultaneously examined audiological characteristics, personality traits, and emotional distress as correlates of tinnitus handicap while including a comparison group of hearing-impaired patients without tinnitus.

The aim of this study was to evaluate the relationship between perceived tinnitus handicap and sociodemographic and clinical characteristics, personality traits, and symptoms of depression, anxiety, and stress. In addition, patients with tinnitus were compared with hearing-impaired patients without tinnitus regarding symptoms of depression, anxiety, and stress. Based on previous findings, we hypothesized that: (H1) tinnitus handicap would be positively associated with depression, anxiety, and stress; (H2) neuroticism would be positively associated with tinnitus handicap; and (H3) psychological variables would explain a greater proportion of variance in tinnitus handicap than sociodemographic or audiological variables.

## 2. Materials and Methods

### 2.1. Patients and Procedures

The study evaluated patients referred to our clinic with a primary complaint of either combined hearing loss and tinnitus, or hearing loss alone. Patients aged 20–60 years were included. Tinnitus duration was required to be at least four months. The exclusion criteria were as follows: age under 20 or over 60 years, objective tinnitus, normal hearing thresholds on pure-tone audiometry, tinnitus duration of less than four months, and a history of previous tinnitus treatment. Patients with vertigo, mental illness, or otological and neurological diseases were also excluded.

The tinnitus group comprised patients suffering from both hearing loss and subjective tinnitus. The control non-tinnitus group comprised patients with hearing loss and no tinnitus. Ethical approval was obtained from the Ethics Committee of the University Clinical Hospital Mostar (No. 85/15, 8 January 2015) and the School of Medicine, University of Mostar (No. 1309/23, 11 April 2023).

The patients had undergone an otological medical examination and pure-tone audiometry, but they had not received any specific tinnitus therapy or counselling at the time they completed the questionnaires. After informed consent was obtained, patients completed the Tinnitus Handicap Inventory, the Depression Anxiety Stress Scales, and the Eysenck Personality Questionnaire—Revised Short Form. Sociodemographic data regarding the patients (age, sex, level of formal education) and descriptive data about the tinnitus were collected.

### 2.2. Measuring Instruments

Tinnitus Handicap Inventory (THI). Tinnitus-related handicap was assessed using the Tinnitus Handicap Inventory (THI), a 25-item self-report questionnaire comprising Functional, Emotional, and Catastrophic subscales [10,11]. Total scores range from 0 to 100, with higher scores indicating greater tinnitus-related handicap. The validated Croatian version (THI-HR) was used [12]. In the present sample, internal consistency was excellent (Cronbach’s α = 0.963 for the total score; α = 0.917, 0.934, and 0.783 for the Functional, Emotional, and Catastrophic subscales, respectively).

Depression Anxiety Stress Scales (DASS). Symptoms of depression, anxiety, and stress were assessed using the 42-item Depression Anxiety Stress Scales (DASS) [13]. The Croatian version has demonstrated good psychometric properties [12]. Higher scores indicate greater symptom severity. Cronbach’s alpha coefficients reported for the Croatian version range from 0.82 to 0.91 [14].

Eysenck Personality Questionnaire—Revised Short Form (EPQ-RS). Personality traits were assessed using the 48-item EPQ-RS, which measures Extraversion, Neuroticism, Psychoticism, and the Lie scale [15]. The Croatian version has demonstrated satisfactory psychometric properties and has previously been validated in Croatian-speaking populations [16].

Perceived tinnitus pitch and loudness. Participants also rated the perceived loudness and pitch of their tinnitus using simple four-point subjective rating scales.

### 2.3. Audiological Assessment

Pure-tone audiometry was performed at 250 Hz, 500 Hz, 1 kHz, 2 kHz, 4 kHz, and 8 kHz for both ears using a GSI 61 Clinical Audiometer (Grason-Stadler, Inc., Milford, NH, USA) equipped with Telephonics TDH-50P supra-aural earphones (Telephonics Corporation, Farmingdale, NY, USA) in an audiometric booth (IAC 120a, IAC Acoustics, Bronx, NY, USA).

Normal hearing was defined as having thresholds of 25 dB HL or lower at all tested frequencies in both ears. Hearing loss was defined as a threshold of 26 dB HL or higher at any tested frequency in either ear. The pure-tone average (PTA) was calculated as the mean of eight air-conduction thresholds, obtained across both ears at 0.5, 1, 2, and 4 kHz.

Of the 311 initially screened patients, 65 were excluded. Because some patients met more than one exclusion criterion, a total of 102 reasons for exclusion were recorded: incomplete or incorrectly filled questionnaires (*n* = 6), tinnitus duration shorter than four months (*n* = 40), active ear infections (*n* = 5), previous tinnitus treatment or counselling (*n* = 5), pulsatile tinnitus (*n* = 7), vertigo (*n* = 5), normal hearing thresholds on pure-tone audiometry (*n* = 31), impacted cerumen (*n* = 2), and multiple sclerosis (*n* = 1).

### 2.4. Study Design

This cross-sectional study examined associations between tinnitus handicap and sociodemographic characteristics, audiological variables, personality traits, and symptoms of depression, anxiety, and stress in patients with chronic tinnitus. Hierarchical multiple regression analysis was used to identify independent correlates of tinnitus handicap. In addition, patients with tinnitus were compared with hearing-impaired controls without tinnitus regarding psychological distress.

### 2.5. Statistical Analysis

Sample size estimation was performed using G*Power software (version 3.1.7). The calculation was based on the primary comparison of psychological distress (Depression Anxiety Stress Scales; DASS) between patients with tinnitus and hearing-impaired controls without tinnitus. Assuming a medium effect size (Cohen’s *d* = 0.50), a statistical power of 80%, and a significance level of α = 0.05, the required sample size was estimated at 106 participants per group. Data normality was assessed using the Kolmogorov–Smirnov test. Depending on data distribution, group differences were examined using the independent-samples *t*-test or the Mann–Whitney *U* test, while associations between variables were assessed using Spearman’s rank correlation coefficient. Hierarchical multiple regression analysis was performed to evaluate the independent contribution of sociodemographic, audiological, psychological, and personality variables to the variance in tinnitus handicap. Prior to regression analysis, multicollinearity was assessed using the variance inflation factor (VIF) and tolerance values, indicating no evidence of problematic multicollinearity. Differences in depression, anxiety, and stress scores across tinnitus severity categories were examined using one-way analysis of variance (ANOVA) followed by Scheffé post hoc tests. Effect sizes were expressed as Cohen’s *d*. Because the tinnitus and non-tinnitus groups differed significantly in age and extraversion, analyses of covariance (ANCOVA) were additionally performed to compare depression, anxiety, and stress scores while controlling for these variables. Internal consistency was evaluated using Cronbach’s alpha coefficients, with values ≥ 0.70 considered acceptable. Statistical significance was set at *p* < 0.05. All analyses were performed using SPSS for Windows (Version 13.0; SPSS Inc., Chicago, IL, USA).

## 3. Results

Of the 311 initially recruited participants, 246 met the inclusion criteria and were included in the analyses: 127 patients with tinnitus and 119 hearing-impaired controls without tinnitus. Sociodemographic and clinical characteristics of both groups are presented in Table 1.

The tinnitus and non-tinnitus groups did not differ significantly in sex distribution (χ^2^ = 4.48, *p* = 0.057), pure-tone average (*t* = −0.22, *p* = 0.830), educational level (*t* = −1.82, *p* = 0.071), neuroticism (*t* = 1.85, *p* = 0.066), or psychoticism (*t* = −0.15, *p* = 0.881). Patients with tinnitus were significantly older (*t* = 3.64, *p* = 0.001), whereas patients without tinnitus scored significantly higher on extraversion (*t* = −2.42, *p* = 0.016). Compared with hearing-impaired controls without tinnitus, patients with tinnitus reported significantly higher depression (*U* = 5446.0, *p* < 0.001), anxiety (*U* = 4812.0, *p* < 0.001), and stress (*U* = 6560.5, *p* < 0.001) scores.

### 3.1. Tinnitus Group

There was no statistically significant correlation between age and the total THI score or any THI subscale (all *p* > 0.05). Likewise, no significant sex differences were observed for the total THI score or any THI subscale (Mann–Whitney *U* test, all *p* > 0.05).

Pure-tone average showed weak positive correlations with the total THI score (*r*_s_ = 0.381, *p* < 0.01) and all THI subscales. Educational level was weakly negatively associated with the total THI score and all THI subscales (all *p* < 0.01).

Because 54 participants (42.5%) were unable to rate tinnitus pitch, this variable was excluded from further analyses.

Subjective tinnitus loudness, depression, anxiety, stress, and neuroticism were positively associated with THI scores, whereas extraversion showed weak negative associations. No significant associations were found for tinnitus duration, psychoticism, or the Lie scale (Table 2).

No significant correlations were observed between tinnitus duration and depression (*r*_s_ = 0.015, *p* = 0.863), anxiety (*r*_s_ = 0.022, *p* = 0.809), or stress (*r*_s_ = 0.074, *p* = 0.407).

Subjective tinnitus loudness was positively associated with the total THI score (*r*_s_ = 0.41, *p* < 0.01). However, formal psychoacoustic tinnitus matching was not conducted; therefore, objectively matched tinnitus-intensity levels were not available.

One-way ANOVA demonstrated significant differences in depression (*F*(4,122) = 9.52, *p* < 0.001), anxiety (*F*(4,122) = 14.18, *p* < 0.001), and stress (*F*(4,122) = 11.95, *p* < 0.001) across THI severity categories. Scheffé post hoc tests showed that participants with catastrophic tinnitus had significantly higher depression, anxiety, and stress scores than those with slight, mild, or moderate tinnitus handicap (Table 3).

Hierarchical multiple regression analysis identified subjective tinnitus loudness, neuroticism, and total emotional distress (DASS total score) as significant variables independently associated with tinnitus handicap. Age, sex, hearing loss, tinnitus duration, educational level, extraversion, psychoticism, and the Lie scale were not significantly associated with the total THI score (Table 4).

### 3.2. Tinnitus Group Versus Non-Tinnitus Group

The tinnitus and non-tinnitus groups did not differ significantly in sex distribution, hearing loss, educational level, psychoticism, or neuroticism (all *p* > 0.05). Patients with tinnitus were significantly older (*t* = 3.64, *p* = 0.001), whereas the control group scored higher on extraversion (*t* = –2.42, *p* = 0.016). Cohen’s *d* indicated a moderate effect for age (*d* = 0.46) and a small effect for extraversion (*d* = –0.31). Compared with hearing-impaired controls, patients with tinnitus reported significantly higher depression (*U* = 5446.0, *p* < 0.001), anxiety (*U* = 4812.0, *p* < 0.001), and stress (*U* = 6560.5, *p* < 0.001) scores (Table 5).

**Figure 1:**
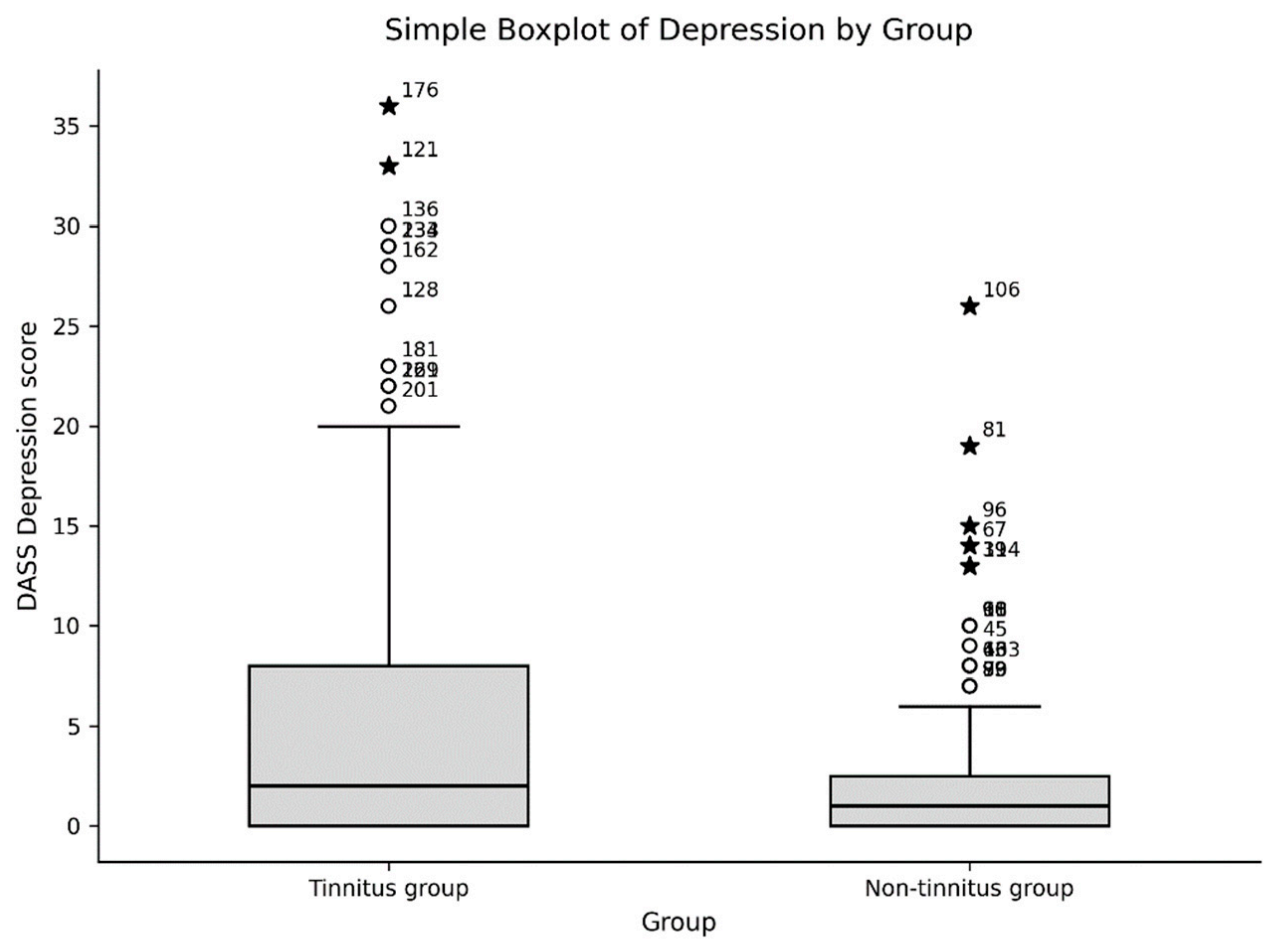
Boxplot showing the distribution of depression scores in the tinnitus and non-tinnitus groups. The box represents the interquartile range, the horizontal line within the box indicates the median, and the whiskers indicate the range of the data excluding outliers. Open circles (○) indicate outliers, whereas stars (★) indicate extreme outliers according to the default SPSS boxplot convention. The numbers displayed next to these symbols represent participant case identification numbers automatically generated by SPSS.

**Figure 2:**
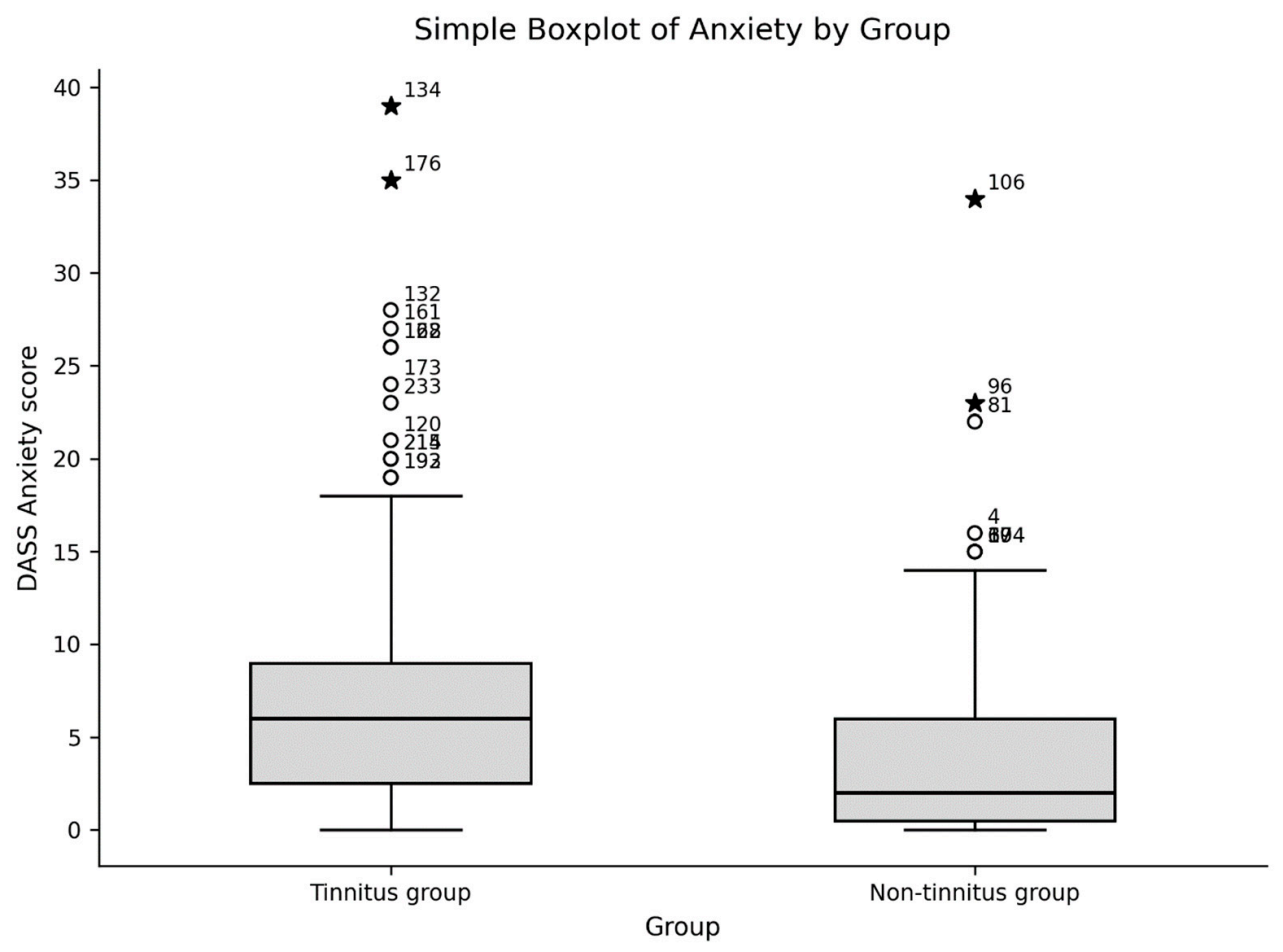
Boxplot showing the distribution of anxiety scores in the tinnitus and non-tinnitus groups. The box represents the interquartile range, the horizontal line within the box indicates the median, and the whiskers indicate the range of the data excluding outliers. Open circles (○) indicate outliers, whereas stars (★) indicate extreme outliers according to the default SPSS boxplot convention. The numbers displayed next to these symbols represent participant case identification numbers automatically generated by SPSS.

**Figure 3:**
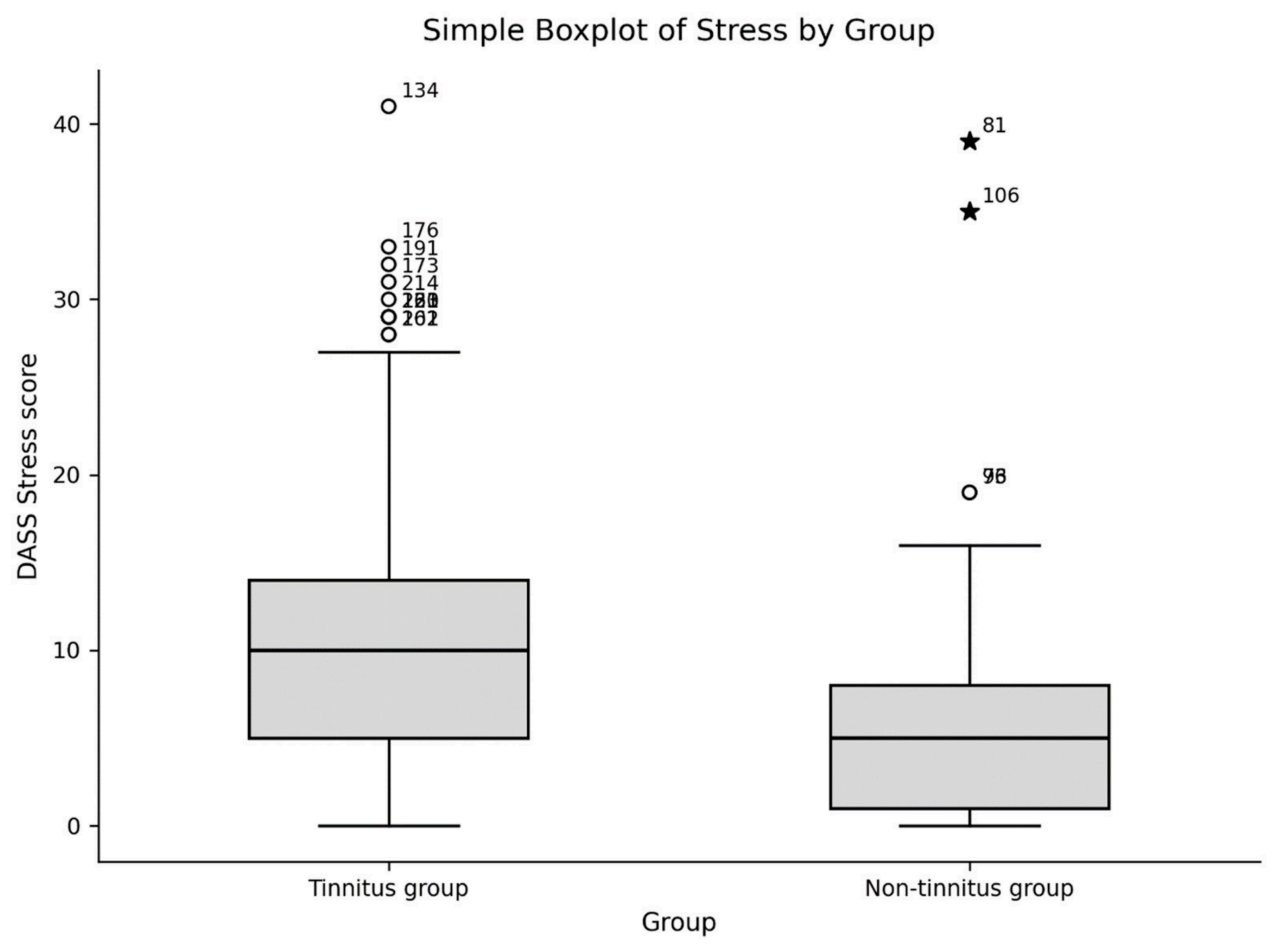
Boxplot showing the distribution of stress scores in the tinnitus and non-tinnitus groups. The box represents the interquartile range, the horizontal line within the box indicates the median, and the whiskers indicate the range of the data excluding outliers. Open circles (○) indicate outliers, whereas stars (★) indicate extreme outliers according to the default SPSS boxplot convention. The numbers displayed next to these symbols represent participant case identification numbers automatically generated by SPSS.

In addition to the DASS severity categories presented in Table 5, the distributions of depression, anxiety, and stress scores in the tinnitus and non-tinnitus groups are shown in Figure 1, Figure 2 and Figure 3, respectively.

Because the groups differed significantly in age and extraversion, additional ANCOVA analyses were performed. After adjustment for these variables, the tinnitus group continued to demonstrate significantly higher levels of depression (*F*(1,242) = 11.14, *p* = 0.001), anxiety (*F*(1,242) = 16.49, *p* < 0.001), and stress (*F*(1,242) = 22.63, *p* < 0.001) than the non-tinnitus group.

## 4. Discussion

The present study investigated the relationships between tinnitus handicap, audiological characteristics, personality traits, and emotional distress in patients with chronic tinnitus. Three principal findings emerged. First, tinnitus handicap was associated with subjective tinnitus loudness, hearing loss, and educational level, although only subjective tinnitus loudness remained independently associated with tinnitus handicap in the multivariable analysis.

The negative association between educational level and tinnitus handicap observed in the present sample should be interpreted cautiously because previous findings have been inconsistent. A recent study reported higher Functional THI subscale scores among participants with primary education and differences in anxiety and depression according to educational level, but found no significant association between education and the total THI score [17]. These discrepancies may reflect differences in sample composition, socioeconomic context, access to healthcare, coping resources, or other unmeasured factors.

Second, neuroticism and emotional distress were independently associated with tinnitus handicap. Third, compared with hearing-impaired controls, patients with tinnitus reported significantly higher levels of depression, anxiety, and stress. Collectively, these findings support a biopsychosocial understanding of tinnitus, suggesting that tinnitus-related disability reflects the interaction of audiological and psychological factors rather than auditory dysfunction alone.

The present findings regarding audiological characteristics are generally consistent with previous studies. Biswas et al. [9] demonstrated that tinnitus prevalence and severity increase with worsening hearing status, while Devos et al. [18], Chen et al. [19], and Mavrogeni et al. [20] reported positive associations between subjective tinnitus loudness and tinnitus-related handicap. However, Searchfield et al. [21] and Teggi et al. [22] found that objective hearing thresholds contributed less consistently to tinnitus severity. Similarly, although hearing loss was associated with tinnitus handicap in the bivariate analyses of the present study, it was no longer independently associated after adjustment for psychological variables. Instead, subjective tinnitus loudness remained the strongest audiological correlate, suggesting that patients’ subjective perception of tinnitus may be more relevant for tinnitus-related disability than objective hearing measures. This interpretation is consistent with contemporary neurophysiological models emphasizing the interaction between auditory dysfunction and cognitive-emotional processing of tinnitus signals [3,23,24].

An important contribution of the present study concerns personality traits. Neuroticism remained independently associated with tinnitus handicap after adjustment for audiological and sociodemographic variables, supporting previous systematic reviews by van Munster et al. [7] and Bernal-Robledano et al. [8], which identified neuroticism as the personality characteristic most consistently related to tinnitus distress. Likewise, Simões et al. [25] reported that higher neuroticism and lower extraversion were associated with greater tinnitus distress and less favourable longitudinal outcomes. Individuals with higher neuroticism may be more likely to interpret tinnitus as threatening, focus attention on tinnitus-related sensations, and engage in maladaptive cognitive processes such as catastrophizing and rumination, thereby increasing tinnitus-related disability.

The present findings also confirm the close relationship between tinnitus handicap and emotional distress. Depression, anxiety, and stress were positively associated with THI scores, and patients with catastrophic tinnitus reported significantly higher levels of all three symptoms than those with lower tinnitus handicap. These findings are consistent with previous systematic reviews and meta-analyses [4,5,6] and with the findings of Gomaa et al. [26]. Furthermore, compared with hearing-impaired controls without tinnitus, patients with tinnitus demonstrated significantly higher levels of depression, anxiety, and stress even after adjustment for age and extraversion. This finding suggests that psychological distress cannot be explained solely by hearing impairment but is specifically associated with the subjective burden imposed by tinnitus.

Another important contribution of the present study is the simultaneous evaluation of audiological, personality, and emotional variables within a single multivariable model. Hierarchical regression analysis demonstrated that subjective tinnitus loudness, neuroticism, and emotional distress were independently associated with tinnitus handicap, whereas hearing loss, tinnitus duration, demographic variables, and other personality dimensions were not. These findings are consistent with those reported by Strumila et al. [27] and extend previous evidence by demonstrating the relative contribution of these domains within the same analytical framework. From a clinical perspective, the findings support routine psychological assessment in patients with chronic tinnitus and reinforce the value of multidisciplinary management involving otolaryngologists, audiologists, psychologists, and mental health professionals.

Several limitations should be acknowledged. First, the cross-sectional design precludes conclusions regarding the direction of the observed associations. Although neuroticism and emotional distress were independently associated with tinnitus handicap, it is equally plausible that persistent and severe tinnitus contributes to increased depression, anxiety, and stress through its effects on sleep, concentration, everyday functioning, and quality of life. Conversely, individuals with greater emotional vulnerability may perceive tinnitus as more intrusive and distressing. These mechanisms are likely to interact in a bidirectional manner, creating a self-perpetuating cycle in which tinnitus exacerbates psychological distress, while psychological distress further amplifies the perceived severity of tinnitus. Consequently, the present findings should be interpreted as evidence of associations rather than causality. Future longitudinal and intervention studies are required to clarify these temporal relationships and determine whether psychological factors represent antecedents, consequences, or reciprocal correlates of tinnitus-related disability. Such studies may also help determine the relative contribution of interventions targeting tinnitus perception, such as Tinnitus Retraining Therapy or bimodal neuromodulation, and psychological interventions, including cognitive-behavioural therapy.

Additional limitations include the single-centre design, which may limit generalizability, and the use of self-report questionnaires, which may be influenced by response bias and shared method variance. Furthermore, although the DASS is a well-validated measure of emotional symptoms, it does not provide clinical psychiatric diagnoses. Personality was assessed using the EPQ-RS, which captures broad personality dimensions but does not evaluate other potentially relevant constructs such as resilience, coping strategies, or psychological flexibility. Finally, the exclusion of patients with previously diagnosed psychiatric disorders may have reduced variability in psychological symptoms.

Another limitation is that formal psychoacoustic tinnitus matching was not performed; therefore, tinnitus pitch and intensity were based on subjective ratings rather than frequency- and intensity-matched measurements. In addition, a normal pure-tone audiogram (≤25 dB HL up to 8 kHz) effectively rules out conventional sensorineural hearing loss; however, it does not guarantee that the auditory system is entirely undamaged [28]. Patients with normal hearing thresholds on pure-tone audiometry were excluded from our study. They did not undergo advanced neuroaudiometric or electrophysiological testing.

Despite these limitations, the present study provides novel evidence from Bosnia and Herzegovina by simultaneously examining audiological characteristics, personality traits, emotional distress, and a hearing-impaired control group without tinnitus. The findings demonstrate that subjective tinnitus loudness, neuroticism, and emotional distress are more strongly associated with tinnitus handicap than objective hearing loss, emphasizing the importance of integrating psychological assessment into routine tinnitus care. Overall, the results support a biopsychosocial model of tinnitus and highlight the need for multidisciplinary approaches that address both auditory and psychological aspects of the condition.

## 5. Conclusions

Subjective tinnitus loudness, neuroticism, and emotional distress were independently associated with tinnitus handicap, whereas objective hearing loss did not remain significant in the multivariable model. Patients with tinnitus also reported greater depression, anxiety, and stress than hearing-impaired controls without tinnitus. These findings support a biopsychosocial understanding of tinnitus and indicate that clinical assessment should integrate audiological evaluation with psychological screening. Because of the cross-sectional design, the findings represent associations rather than causal relationships, and longitudinal and intervention studies are needed to clarify their direction.

## Figures and Tables

**Table 1:** Sociodemographic and clinical characteristics of patients with tinnitus and hearing-impaired controls without tinnitus.

Characteristic	Tinnitus Group (*N* = 127)	Non-Tinnitus Group (*N* = 119)	Test Statistic	*p*-Value
Age, years, mean ± SD	49.00 ± 9.14	44.62 ± 10.65	*t* = 3.64	**0.001**
Sex, *n* (%)			χ^2^ = 4.48	0.057
Male	81 (63.8)	60 (50.4)	—	—
Female	46 (36.2)	59 (49.6)	—	—
Educational level, *n* (%)			*t* = −1.82	0.071
Primary school	5 (3.9)	0 (0.0)	—	—
Secondary school	87 (68.5)	70 (58.8)	—	—
College	10 (7.9)	27 (22.7)	—	—
University	25 (19.7)	22 (18.5)	—	—
Tinnitus duration, months, median [IQR], min–max	24 [50.5], 4–360	N/A	N/A	N/A
Pure-tone average, dB HL, mean ± SD	29.40 ± 13.83	29.04 ± 12.20	*t* = −0.22	0.830
Tinnitus location, *n* (%)		N/A	N/A	N/A
Right ear	27 (21.3)	—	—	—
Left ear	41 (32.3)	—	—	—
Both ears	45 (35.4)	—	—	—
Inside the head	7 (5.5)	—	—	—
Unable to determine	7 (5.5)	—	—	—
Tinnitus manifestation, *n* (%)		N/A	N/A	N/A
Intermittent	36 (28.3)	—	—	—
Constant	91 (71.7)	—	—	—
Subjective tinnitus loudness, *n* (%)		N/A	N/A	N/A
Very faint	3 (2.4)	—	—	—
Faint	20 (15.7)	—	—	—
Moderate	82 (64.6)	—	—	—
Very loud	22 (17.3)	—	—	—
Subjective tinnitus pitch, *n* (%)		N/A	N/A	N/A
Low	27 (21.3)	—	—	—
High	46 (36.2)	—	—	—
Unable to determine	54 (42.5)	—	—	—
THI severity category, *n* (%)		N/A	N/A	N/A
Slight or no handicap	44 (34.6)	—	—	—
Mild handicap	42 (33.1)	—	—	—
Moderate handicap	16 (12.6)	—	—	—
Severe handicap	12 (9.4)	—	—	—
Catastrophic handicap	13 (10.2)	—	—	—
Depression, median [IQR], min–max ^†^	2 [8], 0–36	1 [3], 0–26	*U* = 5446.0	**<0.001**
Anxiety, median [IQR], min–max ^†^	6 [7], 0–39	2 [6], 0–34	*U* = 4812.0	**<0.001**
Stress, median [IQR], min–max ^†^	10 [9], 0–41	5 [7], 0–39	*U* = 6560.5	**<0.001**
Extraversion, mean ± SD ^‡^	8.05 ± 2.80	8.85 ± 2.35	*t* = −2.42	**0.016**
Neuroticism, mean ± SD ^‡^	4.74 ± 3.56	3.92 ± 3.38	*t* = 1.85	0.066
Psychoticism, mean ± SD ^‡^	3.06 ± 1.58	3.09 ± 1.55	*t* = −0.15	0.881

Note: Data are presented as mean ± standard deviation (SD), median [interquartile range (IQR)], minimum–maximum, or number and percentage, *n* (%), as appropriate. Between-group comparisons were performed using independent-samples *t*-tests, Mann–Whitney *U* tests, or Pearson’s chi-square test, as appropriate. Tinnitus-specific characteristics were recorded only in the tinnitus group and were therefore not included in between-group comparisons. Statistically significant *p*-values are shown in bold. Statistical significance was set at *p* < 0.05. THI, Tinnitus Handicap Inventory; DASS, Depression Anxiety Stress Scales; EPQ-RS, Eysenck Personality Questionnaire—Revised Short Form; dB HL, decibels hearing level; N/A, not applicable. ^†^ Measured using the DASS. ^‡^ Measured using the EPQ-RS.

**Table 2:** Spearman’s rank correlation coefficients of Tinnitus Handicap Inventory scores with subjective tinnitus ratings, Depression Anxiety Stress Scales (DASS), and Eysenck Personality Questionnaire—Revised Short Form (EPQ-RS) scores (*N* = 127).

	THI Total *r_s_*	Functional *r_s_*	Emotional *r_s_*	Catastrophic *r_s_*
Duration of tinnitus (months)	0.02	0.06	0.02	0.03
Tinnitus loudness	0.41 **	0.35 **	0.38 **	0.40 **
DASS (Depression)	0.50 **	0.46 **	0.49 **	0.47 **
DASS (Anxiety)	0.55 **	0.51 **	0.51 **	0.51 **
DASS (Stress)	0.57 **	0.53 **	0.55 **	0.52 **
EPQ-RS (Extraversion)	−0.25 **	−0.20 *	−0.26 **	−0.24 **
EPQ-RS (Neuroticism)	0.56 **	0.54 **	0.56 **	0.46 **
EPQ-RS (Psychoticism)	0.05	0.05	0.04	0.08
EPQ-RS (Lie)	−0.14	−0.16	−0.13	−0.05

* Statistically significant (*p* < 0.05); ** statistically significant (*p* < 0.01).

**Table 3:** Multiple comparisons (Scheffé post-hoc test) of Depression, Anxiety, and Stress Scales scores across the five Tinnitus Handicap Inventory (THI) severity categories.

THI Category	Comparison	Depression Subscale *p*-Value	Anxiety Subscale *p*-Value	Stress Subscale *p*-Value
Slight handicap	Mild handicap	0.75	0.12	0.20
	Moderate handicap	0.99	0.45	0.60
	Severe handicap	0.004 *	0.01 *	0.02 *
	Catastrophic handicap	<0.001 *	<0.001 *	<0.001 *
Mild handicap	Slight handicap	0.75	0.12	0.20
	Moderate handicap	0.99	0.65	0.70
	Severe handicap	0.07	0.08	0.09
	Catastrophic handicap	<0.001 *	<0.001 *	<0.001 *
Moderate handicap	Slight handicap	0.99	0.45	0.60
	Mild handicap	0.99	0.65	0.70
	Severe handicap	0.07	0.06	0.08
	Catastrophic handicap	<0.001 *	<0.001 *	<0.001 *
Severe handicap	Slight handicap	0.004 *	0.01 *	0.02 *
	Mild handicap	0.07	0.08	0.09
	Moderate handicap	0.07	0.06	0.08
	Catastrophic handicap	0.13	0.10	0.12
Catastrophic handicap	Slight handicap	<0.001 *	<0.001 *	<0.001 *
	Mild handicap	<0.001 *	<0.001 *	<0.001 *
	Moderate handicap	<0.001 *	<0.001 *	<0.001 *
	Severe handicap	0.13	0.10	0.12

* Statistically significant (*p* < 0.05).

**Table 4:** Hierarchical multiple regression analysis of predictors of the Tinnitus Handicap Inventory total score.

Predictors	Model 1 B	*p*	Model 2 B	*p*	Model 3 B	*p*
Age	−0.12	0.646	−0.25	0.269	−0.16	0.485
Sex	−1.12	0.813	−1.04	0.797	−0.94	0.813
Hearing loss	−0.00	0.545	−0.00	0.611	−0.00	0.448
Tinnitus duration	0.04	0.241	0.03	0.231	0.03	0.245
Tinnitus loudness	15.17	<0.001	11.86	<0.001	11.96	**<0.001**
Duration of education	−2.92	0.021	−1.50	0.168	−1.37	0.201
Extraversion	—	—	0.30	0.704	0.45	0.559
Neuroticism	—	—	4.07	<0.001	2.63	**0.002**
Psychoticism	—	—	−0.87	0.483	−0.82	0.501
Lie scale	—	—	0.22	0.772	0.06	0.932
DASS-total ^†^	—	—	—	—	0.31	**0.016**
*R* ^2^	0.210		0.457		0.483	
Δ*R*^2^	—		0.246		**0.027**	
*F* model	5.33 ***		9.75 ***		9.79 ***	

B = unstandardized regression coefficient; *R*^2^ = coefficient of determination; Δ*R*^2^ = change in the coefficient of determination; *** *p* < 0.001; ^†^ the Depression Anxiety Stress Scales.

**Table 5:** Comparison of Depression, Anxiety, and Stress Scale scores between patients with and without tinnitus.

	Tinnitus Patients (*N* = 127)	Tinnitus-Free Patients (*N* = 119)	Tinnitus Patients vs. Tinnitus-Free Patients *p*-Value ^‡^
Depression subscale: severity grades (normal/mild/moderate/severe/extremely severe) (*n*) ^†^	106/8/2/5/6	110/6/2/1/0	
Depression: median [IQR] ^†^	2 [8]	1 [3]	0.001 *
Anxiety subscale: severity grades (normal/mild/moderate/severe/extremely severe) (*n*) ^†^	83/14/11/8/11	94/13/5/4/3	
Anxiety: median [IQR] ^†^	6 [7]	2 [6]	0.001 *
Stress subscale: severity grades (normal/mild/moderate/severe/extremely severe) (*n*) ^†^	99/11/3/12/2	110/5/2/0/2	
Stress: median [IQR] ^†^	10 [9]	5 [7]	0.001 *

* Statistically significant (*p* < 0.01); ^†^ measured by the Depression Anxiety Stress Scales; ^‡^ Mann–Whitney *U* test.

## Data Availability

The original contributions presented in this study are included in the article. Further inquiries can be directed to the corresponding author(s).

## References

[1] Mavrogeni P., Maihoub S., Molnár A. (2025). Speech recognition thresholds correlate with tinnitus intensity in individuals with primary subjective tinnitus. Front. Neurol..

[2] Molnár A., Mavrogeni P., Mavrogenis A., Maihoub S. (2026). Levels of 25-hydroxyvitamin D_3_ in individuals with primary subjective tinnitus and their associations with tinnitus occurrence and severity. Front. Neurol..

[3] Jastreboff P.J., Hazell J.W. (1993). A neurophysiological approach to tinnitus: Clinical implications. Br. J. Audiol..

[4] Meijers S.M., Rademaker M., Meijers R.L., Stegeman I., Smit A.L. (2022). Correlation between chronic tinnitus distress and symptoms of depression: A systematic review. Front. Neurol..

[5] Hackenberg B., Döge J., O'Brien K., Bohnert A., Lackner K.J., Beutel M.E., Michal M., Münzel T., Wild P.S., Pfeiffer N. (2023). Tinnitus and its relation to depression, anxiety, and stress—A population-based cohort study. J. Clin. Med..

[6] Jiang Y., Liu Q., Ding Y., Sun Y. (2025). Systematic review and meta-analysis of the correlation between tinnitus and mental health. Am. J. Otolaryngol..

[7] van Munster J.J.C.M., van der Valk W.H., Stegeman I., Lieftink A.F., Smit A.L. (2020). The relationship of tinnitus distress with personality traits: A systematic review. Front. Neurol..

[8] Bernal-Robledano A., Parra-Perez A.M., Moleon M.D.C., Lopez-Escámez J.A., Perez-Carpena P. (2025). Personality traits associated with tinnitus: A systematic review and contributing genetic variants. Neurosci. Biobehav. Rev..

[9] Biswas R., Lugo A., Akeroyd M.A., Schlee W., Gallus S., Hall D.A. (2021). Tinnitus prevalence in Europe: A multi-country cross-sectional population study. Lancet Reg. Health Eur..

[10] Newman C.W., Sandridge S.A., Jacobson G.P. (1998). Psychometric adequacy of the Tinnitus Handicap Inventory (THI) for evaluating treatment outcome. J. Am. Acad. Audiol..

[11] McCombe A., Baguley D., Coles R., McKenna L., McKinney C., Windle-Taylor P. (2001). Guidelines for the grading of tinnitus severity: The results of a working group commissioned by the British Association of Otolaryngologists, Head and Neck Surgeons, 1999. Clin. Otolaryngol. Allied Sci..

[12] Jelavić B., Čarapina Zovko I., Pehar I., Sušac T., Lesko J., Leventić M. (2025). Croatian Version of Tinnitus Handicap Inventory—THI-HR. Acta Clin. Croat..

[13] Parkitny L., McAuley J. (2010). The Depression Anxiety Stress Scales (DASS). J. Physiother..

[14] Reić Ercegovac I., Penezić Z., Proroković A., Ćubela Adorić V., Penezić Z., Tucak Junaković I. (2012). Skala Depresivnosti, Anksioznosti i Stresa (DASS). Zbirka psihologijskih skala i upitnika, Svezak 6.

[15] Eysenck S.B.G., Eysenck H.J., Barrett P. (1985). A revised version of the psychoticism scale. Pers. Individ. Dif..

[16] Ivković V., Vitart V., Rudan I., Janićijević B., Smolej-Narančić N., Škarić-Jurić T., Barbalic M., Polasek O., Kolcic I., Biloglav Z. (2007). The Eysenck personality factors: Psychometric structure, reliability, heritability and phenotypic and genetic correlations with psychological distress in an isolated Croatian population. Pers. Individ. Dif..

[17] Molnár A., Mavrogeni P., Maihoub S. (2026). Impact of education, sex, and residence on tinnitus distress, depression, and anxiety. Audiol. Res..

[18] Devos J.V.P., Janssen M.L.F., Janssen A.M.L., Hellingman C.A., Smit J.V. (2024). A prospective self-report survey-based cohort study on factors that have an influence on tinnitus. Audiol. Res..

[19] Chen S., Shen X., Yuan J., Wu Y., Li Y., Tong B., Qiu J., Wu F., Liu Y. (2022). Characteristics of tinnitus and factors influencing its severity. Ther. Adv. Chronic Dis..

[20] Mavrogeni P., Molnár A., Molnár V., Tamás L., Maihoub S. (2024). Correlation between the pitch and loudness of tinnitus, hearing levels, and Tinnitus Handicap Inventory scores in patients with chronic subjective tinnitus. J. Clin. Med..

[21] Searchfield G.D., Jerram C., Wise K., Raymond S. (2007). The impact of hearing loss on tinnitus severity. Aust. N. Z. J. Audiol..

[22] Teggi R., Cangiano I., Familiari M., Gioffrè V., Nobile A., Gatti O. (2025). The Tinnitus Handicap Inventory total score: What really counts? Experience on a sample of 1156 patients. Audiol. Res..

[23] Cima R.F., Maes I.H., Joore M.A., Scheyen D.J., El Refaie A., Baguley D.M., Anteunis L.J., van Breukelen G.J., Vlaeyen J.W. (2012). Specialised treatment based on cognitive behaviour therapy versus usual care for tinnitus: A randomised controlled trial. Lancet.

[24] Monzani D., Genovese E., Marrara A., Gherpelli C., Pingani L., Forghieri M., Rigatelli M., Guadagnin T., Arslan E. (2008). Validity of the Italian adaptation of the Tinnitus Handicap Inventory; focus on quality of life and psychological distress in tinnitus-sufferers. Acta Otorhinolaryngol. Ital..

[25] Simões J., Schlee W., Schecklmann M., Langguth B., Farahmand D., Neff P. (2019). Big five personality traits are associated with tinnitus improvement over time. Sci. Rep..

[26] Gomaa M.A., Elmagd M.H., Elbadry M.M., Kader R.M. (2014). Depression, Anxiety and Stress Scale in patients with tinnitus and hearing loss. Eur. Arch. Otorhinolaryngol..

[27] Strumila R., Lengvenytė A., Vainutienė V., Lesinskas E. (2017). The role of questioning environment, personality traits, depressive and anxiety symptoms in tinnitus severity perception. Psychiatr. Q..

[28] Schaette R., McAlpine D. (2011). Tinnitus with a normal audiogram: Physiological evidence for hidden hearing loss and computational model. J. Neurosci..

